# Feeding behaviour, risk-sensitivity and response control: effects of 5-HT_2C_ receptor manipulations

**DOI:** 10.1098/rstb.2018.0144

**Published:** 2018-12-31

**Authors:** Trevor Humby, Yateen Patel, Jenny Carter, Laura-Jean G. Stokes, Robert D. Rogers, Lawrence S. Wilkinson

**Affiliations:** 1 Behavioral Genetics Group, Cardiff University, Cardiff CF10 3AX, UK; 2 MRC Centre for Neuropsychiatric Genetics and Genomics, Cardiff University, Cardiff CF10 3AX, UK; 3 Division of Psychological Medicine and Clinical Neurosciences, Cardiff University, Cardiff CF10 3AX, UK; 4 Neuroscience and Mental Health Research Institute, Cardiff University, Cardiff CF10 3AX, UK; 5 School of Psychology, Bangor University, Bangor LL57 2AS, UK

**Keywords:** feeding, risk sensitivity, response control, 5-HT_2C_R, SB242084, WAY161503

## Abstract

People, like animals, tend to choose the variable option when given the choice between a fixed and variable delay to reward where, in the variable delay condition, some rewards are available immediately (Laura-Jean *et al*. 2019 *Phil. Trans. R. Soc. B* **374**, 20180141. (doi:10.1098/rstb.2018.0141)). This bias has been suggested to reflect evolutionary pressures resulting from food scarcity in the past placing a premium on obtaining food quickly that can win out against the risks of sometimes sustaining longer delays to food. The psychologies mediating this effect may become maladaptive in the developed world where food is readily available contributing, potentially, to overeating and obesity. Here, we report our development of a novel touchscreen task in mice allowing comparisons of the impact of food delay and food magnitude across species. We show that mice exhibit the typical preference, as shown by humans, for variable over fixed delays to rewards but no preference when it comes to fixed versus variable reward amounts and further show that this bias is sensitive to manipulations of the 5-HT_2C_ receptor, a key mediator of feeding and impulse control. We discuss the data in terms of the utility of the task to model the psychologies and underlying brain mechanisms impacting on feeding behaviours.

This article is part of the theme issue ‘Risk taking and impulsive behaviour: fundamental discoveries, theoretical perspectives and clinical implications’.

## 1. Background

People, like animals, can show ‘risk-seeking’ behaviour for food rewards, i.e. preferring a variable interval schedule of food availability encompassing very short delays interspersed with much longer delays, to a fixed delay that is between the short and long extremes of the variable delay [1]. Although humans, and other species, may be able to plan for famine and cache food for these periods [2,3], finding food during these times of need may involve increased risk for successful foraging. Thus, it has been suggested that risk-seeking behaviour might reflect evolutionary pressures formed as a result of food scarcity which has placed a premium on obtaining food immediately against the risks of sometimes sustaining longer delays to the delivery of food [4,5]. It has also been suggested that the psychologies mediating this effect may become maladaptive in the developed world where food is readily available contributing to overeating and obesity [6], and indeed the response bias to variable delays has been shown to be sensitive to vulnerability factors for weight gain that promote food-seeking behaviour (such as the presence of food cues) [1,4,5].

Rogers *et al.* [7] have speculated that food scheduling of the kind examined in the companion article by Stokes *et al.* [1], and the impact of controlling variables such as the presence of food cues, may overlap with the psychologies and brain mechanisms underlying impulsive responding. Individual differences in response control are mediated predominately by circuits incorporating the prefrontal cortex and striatum, modulated by forebrain neuromodulators [8–10]. In the present context of biases in feeding behaviour, the effects of manipulating the forebrain dopaminergic systems have been shown to influence preferences for ‘risky’ variable delays over ‘less risky’ fixed delays for food reward in rodents [7,11]. Less is known about other neurotransmitter systems, though we have previously shown in rats that variable delay preferences are also diminished by the administration of the 5-HT_1A_R agonist 8-OH-DPAT [7], suggesting roles for both dopaminergic and serotonergic mechanisms in food-scheduling behaviour [8].

One interesting candidate mechanism at the nexus of feeding and response control is the 5-HT_2C_ receptor (5-HT_2C_R). The 5-HT_2C_R is distributed through the brain with expression in the prefrontal cortex, hippocampus, striatum, amygdala and the nuclei of the hypothalamus; regions implicated in both response control [12,13] and eating-related behaviours [14–16]. Deletion of 5-HT_2C_R in animal models is associated with a number of behavioural phenotypes relevant to feeding behaviours [17–19]. 5-HT_2C_R-null mice eat more and become obese in adulthood [20,21] and consistent with this finding selective 5-HT_2C_R agonists decrease appetite [22,23]. Moreover, mouse models of Prader–Willi Syndrome (PWS-IC), a disorder where a main abnormality is a profound overeating [24], display hyperphagia and a reduced effectiveness of the anorexic effects of 5-HT_2C_R agonism, along with effects on cognition [25–27]. These effects are relevant to 5-HT_2C_R functioning because the genetic lesion in PWS-IC mice encompasses a deletion of SNORD115, a small nucleolar RNA molecular involved in 5-HT_2C_R editing and splicing [28], leading to less functional 5-HT_2C_Rs in PWS-IC mice [29]. In tandem with effects on eating, we and others have also shown in both rat and mouse models that manipulation of 5-HT_2C_R function impacts on risk sensitivity and response control across a wide range of experimental settings and tasks, including delayed reinforcement, 5-choice serial reaction time task and stop signal reaction time task [9,10,30,31].

In this paper, we report the development and behavioural specification of an operant task for use in mice informed by the food-scheduling models and methodologies described by Stokes *et al.* [1]. We assayed risk tolerance using a novel touchscreen platform that allowed an assessment of choices between variable delays over fixed delays to reward and also variable versus fixed amounts of reward. Reflecting extensive evidence from foraging and operant contexts in other species [4,5,32–34], we anticipated that mice would demonstrate ‘risky’ preferences for the variable delay to reward [32,33] and this is what we found. The preference for choosing variable delays to reward was shown to be robust following reversal of the stimulus/response contingencies. By contrast, the mice exhibited no preference in terms of fixed or variable reward amounts. We also examined the effects of acute pharmacological manipulations of 5-HT_2C_R function on the preference for variable delays to reward using the selective 5-HT_2C_R antagonist SB242084 and 5-HT_2C_R agonist WAY161503. The pattern of drugs effects was indicative of a high degree of specificity of 5-HT_2C_R action on risk-seeking behaviour for food rewards and was also, we argue, consistent with a predominant effect on the psychologies and brain mechanisms mediating response control.

## 2. Material and methods

### (a) Subjects

A cohort of 44 male C57Bl/6OlaHsd mice (Envigo, UK), two months old at the start of the experiment, was housed in groups of four, in a vivarium (temperature: 21 ± 2°C, humidity 50% ± 10) and a 12 L : 12 D cycle (lights on at 07.00). Food was available *ad libitum* during the experiment. Following two weeks of habituation and handling, the mice were placed on a home cage water restriction schedule to motivate the animals to work. Initially, mice were restricted to 4 h access per day and once bodyweight and drinking have stabilized, this was reduced to 2 h access per day for the remainder of the experiment. Mice were monitored throughout the experiment to check health and well-being, and periodically, the mice were given 48 h of free access to water. Animals were treated in accordance with the Animal (Scientific Procedures) Act (United Kingdom, 1986), and experiments performed under a UK project licence (PPL: 30/3135).

### (b) Apparatus

**Figure 1.**
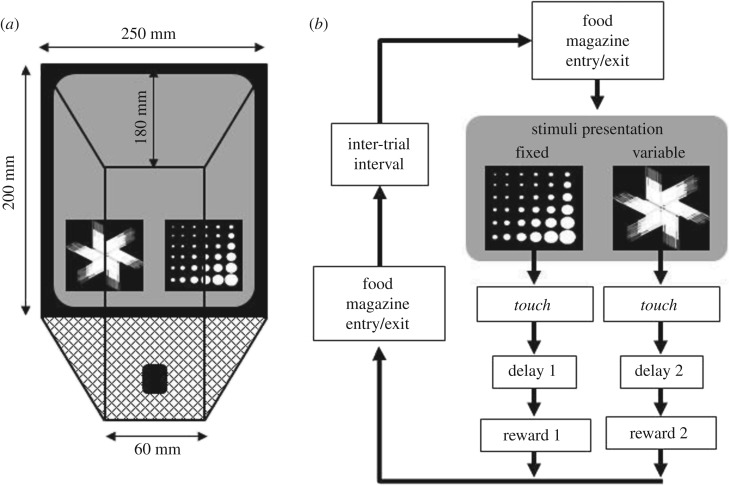
Apparatus and task schedule for the fixed versus variable delay to reward (FST-D) or fixed versus variable reward amount (FST-R) food scheduling tasks. (*a*) The apparatus used was a touchscreen chamber (Campden Cognition, UK), consisting of a trapezoid-shaped animal enclosure with a touch-sensitive screen making up the larger wall. (*b*) A trial started with presentation of two stimuli to the screen, touching either one would lead to reward presentation following a delay. Delay durations in the FST-D task or reward amount in the FST-R task, and for the probe manipulation sessions, were determined by the response made (see figure 2*a*). Different pairs of stimuli were used for each phase of the task and for each new test within each phase (see electronic supplementary material, methods figure S1 for stimuli pairs used).

All testing took place in two systems of four mouse touchscreen operant chambers (Campden Cognition, UK, and see [35]), under the control of custom written software (ABET, Campden Cognition, UK). Each touchscreen chamber was housed within a sound attenuating box. The mouse enclosures were of a trapezoid shape, with Perspex walls on three sides, and a metal grid floor (figure 1*a*). The fourth side consisted of the touchscreen, on which stimuli could be presented and to which the mice were trained to respond. For the current task, involving choices between variable and fixed delays to liquid rewards, the touchscreen was occluded by a black Perspex mask with two 70 mm square response apertures, 20 mm from the grid base of the chamber and positioned equally across the width of the mask. In the opposite side of the chamber, 5 mm from the grid base, was a 20 mm square recess (25 mm deep) into which liquid reward was delivered. Infrared beams were used to record motor activity and an infrared CCTV system permitted observation of the mice.

### (c) Behavioural procedure

Once the mice were stabilized on the water restriction schedule, they were habituated to the reward to be used in the experiment, 10% condensed milk solution, using standardized methods [30], demonstrating greater than or equal to 80% preference for the condensed milk solution versus water. The mice were then habituated to the touchscreen chambers in three daily 20 min sessions in which the reward (22 µl) was delivered every 30 s. Following this phase of training, the mice were next shaped to touch the touchscreen to earn reward: single-stimulus training (SST). In daily 20 min sessions of 60 trials, a single pattern stimulus per trial was randomly presented to one of the stimuli locations on the touchscreen, which remained on screen until touched. Stimuli were randomly selected from the supplied image database. Touching the stimulus initiated the delivery of 22 µl of reward. Mice continued with SST until they completed greater than 50 trials in a session for two consecutive days.

**Figure 2.**
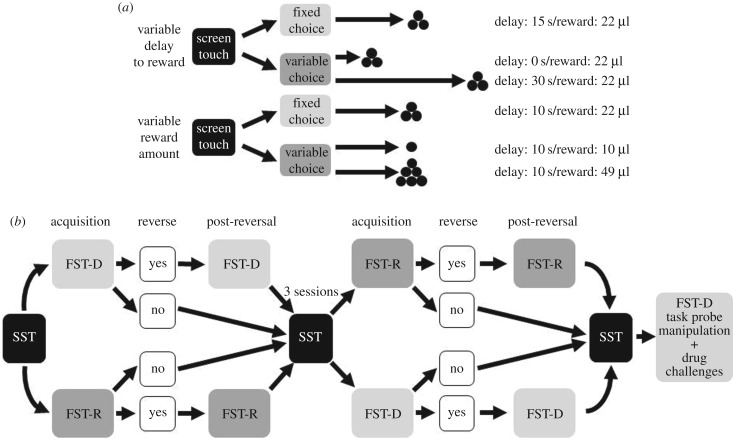
Schematic of the experimental design and procedures. For both the fixed versus variable delay to reward (FST-D) and fixed versus variable reward amount (FST-R) food-scheduling tasks, the subjects were presented with a choice between a fixed option and a variable option, which had two possible outcomes (1 : 1 probability of presentation). The combination of delay durations and reward quantities for each task (*a*) were determined such that the coefficients of reinforcement were equivalent [36,37]. Mice were initially shaped to respond to the touchscreens with presentation of a single stimulus to the screen (SST), before proceeding through the FST-R and FST-D tasks, with task order counter-balanced between subjects (*b*). Between the tasks, the mice were given three transition sessions of SST to stabilize behaviour. Performance criteria for reversal: consistent/stable choice performance (>75% choice preference) for more than two sessions, maximum of 20 sessions per phase.

Once at SST criterion, the mice were moved to the main food-scheduling task (FST) [1] protocol in which two stimuli were now presented to the touchscreen (figure 1*b*), with locations of each stimulus randomly determined per trial. Sessions were 20 min or 60 trials long and were terminated by whichever criterion was reached first. Responding to each stimulus would lead to a reward, but under dissociable conditions (figure 2*a*). Thus, in the fixed versus variable delay to reward version of the FST (FST-D), 22 µl reward was delivered after the selection of either the fixed 15 s delay choice or the variable 0 or 30 s delays (each with a 0.5 probability of presentation) option. In the fixed versus variable reward amount version of the FST (FST-R), the delay to reward delivery was a constant 10 s for both choices, but selection of the fixed option led to the delivery of 22 µl of reward, whereas choosing the alternative could lead to delivery of either 10 or 49 µl (each with a 0.5 probability of presentation). The combination of delay durations and reward quantities for each task was determined such that the coefficients of reinforcement were equivalent [36,38] and that there were no significant advantages in the amount or frequency of the reward between the fixed and variable choices in each task (electronic supplementary material, methods table S1). Stimuli associated with each task choice (fixed or variable) were counter-balanced between subjects.

All mice were assessed in both the FST-D and FST-R procedures, counter-balanced for testing order (figure 2*b*). There were two test phases for each task, initial acquisition and reversal, with a maximum of 20 sessions per phase. Transfer between the phases was based on achieving performance criterion, set as an arbitrary 75% preference for one of the choices in two successive sessions. Mice that failed to reach the preference criterion within the 20 available sessions did not go through the reversal procedure but instead went directly to the alternate task. For those animals that reached the preference criterion during the initial acquisition, the stimulus–reward contingencies were reversed, such that the stimulus that was previously related to the variable delay/reward now became the fixed delay/reward, and vice versa for the other stimulus. Following reversal, these mice were given a further 20 sessions in each task to re-establish stable responding. If they achieved the preference criterion, then they were switched to the other task. Between the two tasks, mice were placed back on the SST for three sessions. On the completion of testing in the main FST-D and FST-R tasks, we determined if the mice would exhibit a preference for one of the stimulus/delay contingencies within a single session FST-D. This was necessary for the acute drug challenge experiments examining the effects of 5-HT_2C_R manipulation on the expression of any choice preferences. Here, performance within the single extended session (30 min/100 trials) was investigated using novel stimuli but with the same delay durations and reward amount as in the FST-D protocol (electronic supplementary material, methods figure S1).

### (d) Pharmacological manipulations

Preferences for fixed or variable delays to reward were assessed following 5-HT_2C_R manipulations with an acute single, extended session/dose-modified FST-D protocol, with new combinations of stimuli in each session (electronic supplementary material, methods figure S1). The 5-HT_2C_R antagonist SB242084 HCl (vehicle, 0.1, 1, 5 mg kg^−1^) and the 5-HT_2C_R agonist WAY161503 HCl (vehicle, 0.1, 1, 5 mg kg^−1^; Tocris, UK) were used as both show high selectivity (pKi = 9.0 and pKi = 7.2, for the 5-HT_2C_R) [37,39]. Drugs were prepared fresh in physiological saline each day, and SB242084 was administered 5 min (subcutaneous, s.c.) and WAY161503 30 min (intraperitoneal, i.p.) prior to the test. Dose ranges were selected based on previous studies in rats and mice using similar operant procedures [9,30,40,41]. In particular, as 5-HT_2C_R agonism at higher doses can produce anorexic effects and decreased locomotor activity [9], we used a dose range of WAY161503 that included lower doses to avoid general disruptions to task performance that may have confounded interpretation of the preference measurements. Each treatment was given using a Latin-square design, with at least 4 days between each dose. Mice were not tested between the drug administration sessions.

### (e) Data analysis

Data were first assessed for normality. The main measure used to gauge performance for the FST-D and FST-R tasks was the preference for the variable versus fixed choice in each task, calculated as a preference ratio: variable choice/(variable choice + fixed choice). Other measures included response latencies for each choice, the time to collect the reward under each condition, the number of completed trials and the number of sessions to reach criterion in each phase of the experiment. Ratios were compared to chance/no preference (0.5) using within subjects *t*-test or equivalent non-parametric analysis such as *Z*, the Wilcoxen-Signed Ranks statistic. The effects of SB242084 and WAY161503 were analysed by separate within-subject ANOVAs with a within-subject factor of dose (vehicle, 0.1, 1, 5 mg kg^−1^). If significant, then *post hoc* pairwise comparisons were performed using Bonferroni correction. Criterion level of significance was set at the 0.05 level. All data are shown as mean ± s.e.m.

## 3. Results

### (a) FST-D and FST-R task acquisition and performance

**Figure 3.**
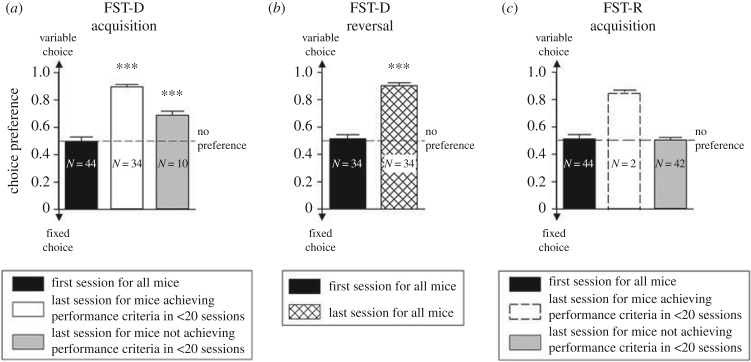
Preference for responding in the fixed versus variable delay to reward (FST-D) and fixed versus variable reward amount (FST-R) food-scheduling tasks. For each task, mice were given a maximum of 20 sessions to reach an arbitrary 75% preference criterion. (*a*) In the FST-D, all mice initially showed no choice preference but a majority (34/44) did reach the preference criterion within the 20 testing sessions available; the mice that did not reach the preference criterion (10/44) still showed a significant above-chance preference. (*b*) The 34 mice that reached the 75% performance criterion in less than 20 sessions in the FST-D task were further tested in reversal. After an initial disruption to behaviour, leading to the temporary abolition of the preference for the variable delay condition, this preference was rapidly re-established post-reversal with all of the mice reaching the 75% preference criterion within 20 sessions. (*c*) In the FST-R task, however, the initial lack of preference for fixed or variable amounts of reward persisted throughout the 20 test sessions available. This was the case for the large majority (42/44) of the animals but two animals did show a preference for the variable amount of reward choice and indeed reached the arbitrary 75% preference criterion, the data for these two mice have been included for completeness. Data show mean ± s.e.m. *N* values for each condition are shown within the individual bar charts. *** denotes *p* < 0.001 for choice preference in comparison to chance (no preference) performance.

Following habituation to the reinforcer and test apparatus, the mice were first shaped to respond to the touchscreen by the presentation of a single stimulus in one of the apertures, counter-balanced across trials. All of the mice demonstrated good responding during this phase of training within approximately eight sessions (electronic supplementary material, results figure S1). In the first session of the FST-D, all the mice showed no initial bias for either the fixed or delay to reward choices (figure 3*a*, *t*_43_ = 0.03, *p* = 0.98). However, within approximately 10 sessions, the majority of mice (34/44) demonstrated a marked preference for the choice that led to a variable delay to reward, relative to chance (figure 3*a*, Z_43_ = 5.09, *p* = 0.0001) and had exceeded the arbitrary criterion of 75% preference. It is important to note that while some animals (10/44) did not manage to reach the 75% preference criterion within the 20 sessions available, these animals still showed above chance preference for the variable delay option (figure 3*a*, Z_43_ = 2.71, *p* = 0.007). Thus, all the animals tested in the FST-D task showed a significant preference for the variable delay condition but there was a degree of individual difference in developing the preference. To test the extent to which it was the contingency between the delays sustained to obtain the fixed amount of reward that was controlling behaviour in the FST-D task, we reversed the choice stimuli. Initially, on reversal behaviour was disrupted with the mice showing no preference for either the fixed or variable delay to rewards (figure 3*b*, *t*_33_ = 0.44, *p* = 0.67) but this effect was short lived and all mice subjected to reversal (34/34) regained the 75% preference criterion for the variable delay choice within approximately 13 sessions, showing a significant bias relative to chance (figure 3*b*, *t*_33_ = 24.11, *p* = 0.0001).

A different pattern of effects was apparent when the mice were tested in the FST-R task. Again, at the beginning of testing, the mice showed no preference for either the fixed or variable reward amount choices (figure 3*c*, *t*_43_ = 0.36, *p* = 0.72) but unlike in the FST-D task, this lack of preference persisted throughout the 20 test sessions available, such that for the large majority of mice (42/44), there was no significant difference in choice preference relative to chance at session 20 (figure 3*c*, *t*_43_ = 0.97, *p* = 0.34). Two mice out of the total of 44 did show a preference for the variable amount choice, reaching the arbitrary 75% preference criterion; the data for these mice have been included in figure 3 for completeness. The inability to clearly discriminate between the fixed and variable reward amount choices led to significant reductions in the completed trials and slowing in choice times between the first and last sessions of the acquisition phase of FST-R testing (electronic supplementary material, results figure S2).

The order of training with the two tasks (FST-D and FST-R), which was counter-balanced between subjects, was without effects on performance and all mice still demonstrated strong preferences for the variable delay choice in the FST-D whether this task came first or second in the run order. Furthermore, as shown in the electronic supplementary material (results figure S3), the performance of the FST-D task was associated with a high degree of stimulus control and motivation as indicated by the number of trials committed per session, the quicker response times and time taken to collect the reward. The main difference in these ancillary measures was an expected reduction in choice times as training progressed which was manifest independently of response choice. There were also no systematic differences in ancillary behavioural measures between those animals that reached the 75% preference criterion and those that did not, and pre- and post-reversal stable performance. Taken together, the pattern of results in choice preference from the two tasks, fixed and variable delay to reward (FST-D) and fixed and variable reward amount (FST-R) were consistent with previous findings in other species [1,4,5,33].

### (b) Drug manipulations

Prior to the drug challenges, we confirmed that the mice could learn to discriminate between the fixed and variable delays to reward in the FST-D task using new stimuli in a single session protocol. In this part of the study, we confined testing to the 34 mice that had shown robust preferences for variable delay to reward in the main FST-D task as indexed by reaching the 75% preference criterion within the 20 available sessions. Under the single session protocol, the mice showed a preference of 0.67 ± 0.02 for the variable delay to reward choice over the fixed delay to reward. However, this choice preference, though significantly above chance (*t*_33_ = 7.88, *p* = 0.0001) was smaller than the typical 0.8–0.9 preference ratios seen in the main FST-D task with multiple training sessions. The relative reduction in the choice preference in the single session FST-D protocol was unsurprising, because the mice had more limited opportunity to manifest a preference with novel stimuli/reward combinations, and consequently fewer mice (13/34) reached a 75% preference criterion, but it was deemed sufficient for the effects of drugs to be analysed.

**Figure 4.**
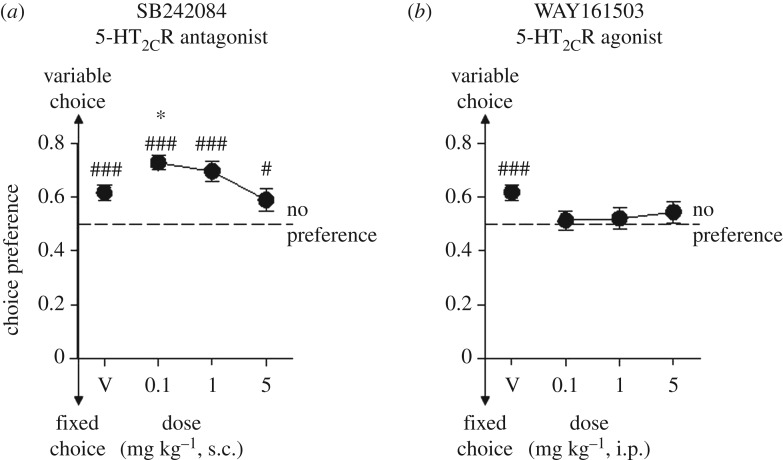
Effects of 5-HT_2C_R antagonism and agonism on the preference for variable or fixed delays to reward. Data showing the effects of increasing doses of (*a*) the selective 5-HT_2C_R antagonist SB212084 and (*b*) selective 5-HT_2C_R agonist WAY161503 on choice preference for fixed or variable delays to reward compared to vehicle (0.9% saline). Choice preferences were assessed following 5-HT_2C_R manipulations with an acute single, extended session/dose-modified FST-D protocol, with new combinations of stimuli for each drug dose. SB212084 was administered s.c. and WAY161503 i.p. Data are mean ± s.e., *N* = 34. * denotes *p* < 0.05 for comparison between vehicle and drug dose, and ^###^ and ^#^ denote *p* < 0.001 and *p* < 0.05 for comparison to chance (no preference) performance.

The effects of the 5-HT_2C_R antagonist SB242084 and 5-HT_2C_R agonist WAY161503 compared to administration of 0.9% vehicle on choices for fixed and variable delay to reward (FST-D) are shown in figure 4. Importantly, following administration of vehicle alone, the preference for the variable delay to reward was significantly above chance (*t*_33_ = 4.23, *p* = 0.0001) at 0.62 ± 0.03, indicating minimal effects of the injections *per se*. The 5-HT_2C_R antagonist SB242084 increased preference for the variable delay choice, relative to vehicle, at the lowest 0.1 mg kg^−1^ dose only (figure 4*a*, main effect of dose, *F*_2.3,77.4_ = 3.91, *p* = 0.02; *p* = 0.02 for *post hoc* comparison between 0.01 mg kg^−1^ dose and vehicle, *p* > 0.05 for other doses relative to vehicle) but at all doses the preference for the variable delay to reward was maintained above chance (*p* < 0.001 for 0.1 and 1 mg kg^−1^ and *p* < 0.05 for 5 mg kg^−1^). The effects of SB242084 were specific to choice preference and were not associated with any drug-induced effects on general features of behaviour across all doses of the drug, such as numbers of trials, response times and the latency to collect the reward (electronic supplementary material, results figure S4). Agonism of the 5-HT_2C_R with WAY161503 was associated with a different pattern of effects, not enhancing the preference for the variable delay choice relative to vehicle-treated animals (which showed a significant above-chance preference for variable delays) but instead at all doses tending to reduce the preference to chance levels (figure 4*b*, main effect of dose, *F*_2.8,94.09_ = 2.17, *p* = 0.09). The two lowest doses of WAY161503 were without effects on ancillary behavioural measures but the highest, 5 mg kg^−1^, dose did lead to significant reductions in the number of trials completed, choice latency and the time taken to collect the reward (electronic supplementary material, results figure S4) which may be due to specific effects, such as anorexic effects or reduced locomotor activity at this dose of drug.

## 4. Discussion

Here, we describe a touchscreen-based model of the concurrent choice procedure based on Stokes *et al.* [1] FST to investigate risk tolerance in the context of feeding behaviour in mice. To our knowledge, this is the first demonstration using an operant approach that this species, like humans [1] and birds [4,5,32], shows an inherent preference for ‘risky’ variable delays over fixed delay schedules [4]. Importantly, the bias towards variable delays was sufficiently strong and flexible to be still present following reversal of the stimuli/delay contingencies, indicating the robustness of the preference. We also describe novel pharmacological data implicating an important role for 5-HT_2C_R mechanisms in mediating risk tolerance towards variable delays in the delivery of rewarding foodstuffs. Together, our findings indicate the utility of the murine touchscreen model system for making valid cross-species comparisons [31,35] and open up the area to the wide range of genetic models available in the mouse.

Consistent with previous data in other animal species, the mice displayed different choice biases depending on the food availability schedules; as noted above, exhibiting risk-seeking behaviour in the context of delays to rewards but no discernible preference between fixed or variable reward amounts [4,34]. To ascertain the controlling variables in the task, we carried out a probe test in which we reversed the stimulus/reward-delay contingencies in the fixed or variable delay to reward food-scheduling task (FST-D). Following a period of adjustment, the animals showed the same strong choice preference for variable delays as before the reversal, indicating that the main variable controlling performance in the task was the impact of the variable/fixed delays. Overall, the data confirmed that the mice were able to learn the task contingencies with a high degree of stimulus control as indicated by the consistent numbers of trials committed per session, and the rapid response latencies and time taken to collect the reward.

Systemic administration of SB242084, a selective 5-HT_2C_R antagonist, increased further the baseline (non-drugged) propensity of the mice to choose the variable delay option versus a fixed delay to reward option in the FST-D task, whereas systemic WAY161503, a selective agonist, tended to reduce the preference for the variable delay to reward option. In order to assess the effects of the drugs on the development and expression of choice preferences in the FST-D task, we employed a single extended testing session using novel stimuli/delay combinations. As noted previously, while this modified protocol did still reveal a significant preference for variable delays in the mice, the extent of the preference was less than that seen in the main multi-session protocol. This is of potential relevance to the interpretation of the drug effects since, while the degree of preference shown by the vehicle-administered animals allowed an increase in preference for the variable delay choice to be seen following treatment with SB242084, it may have compressed the range available to detect the decrease in preference observed with WAY161503. However, this effect was not a true floor effect because WAY161503 did not elicit a switch over to the fixed delay option which the animals were perfectly free to do but instead reduced responding to chance. Nonetheless, some degree of caution should be attached to these data and it would be interesting, in future work, to also assess the effects of 5-HT_2C_R antagonism and agonism at performance as well as during the acquisition of choice preferences. These new data on 5-HT_2C_R mechanisms add to the previous finding that administration of the 5-HT_1A_R agonist, 8-OH-DPAT, dose-dependently diminished selections of variable delay to reward over fixed delay to reward options [7] at performance and extend the influence of the serotonin system on risk tolerance in the context of feeding.

Our observation that SB242084 and WAY161503 produce opposing effects upon the preference for variable over fixed delay schedules could potentially reflect actions within two basic functional domains of the 5-HT_2C_R system: effects on appetite and drive to eat *per se* and/or changes in delay discounting, response control functions. As noted earlier, 5-HT_2C_Rs have well established roles in appetite and satiety; in particular, 5-HT_2C_R agonism is known to cause anorexic responses in rodents. However, without discounting them totally it is difficult to envisage that drug effects on feeding *per se* were a main driver of the behaviour because such general effects cannot easily be accommodated with the pattern of results on choice preferences. Moreover, there were no indications from ancillary aspects of behaviour, such as response latencies and the latency to collect reward, for effects related to changes in appetite, satiety or hedonic characteristics of the food reward at any of the doses of SB242084 used. This was also the case for the two lower doses of WAY161503. However, as noted in the Results, the highest 5 mg kg^−1^ dose did lead to significant reductions in the number of trials completed, choice latency and the time taken to collect the reward, which may be related to the previously reported effects on motor activity patterns [9]. Here, the possibility of known anorexic effects of WAY161503 [40] influencing choice preferences at this dose might be entertained.

On the other hand, the opposing effects of agonist and antagonists of 5-HT_2C_R on choice preference may reflect some modulation of impulse control. These could arise in two ways. First, preferences for variable over fixed delays schedules have been hypothesized to reflect the greater summed value of immediate and discounted rewards delivered following longer delays compared with the single moderately discounted rewards delivered following intermediate rewards ([5,32–34] but see [38]). From this view, the opposing effects of SB242084 and WAY161503 administration upon preferences for variable over fixed delay to reward schedules could represent changes in the values of discounted rewards. A second interpretation of the drug effects is related to data showing that the 5-HT_2C_R antagonist, SB242084, can increase premature responding in operant settings [9,10]. Possibly therefore, especially under conditions of mild water deprivation, the subjects of our experiment acquired preferences for the variable delay schedule as the pre-potent option that offered the possibility of immediate liquid rewards but at the risk of longer delays than the intermediate delays offered by the fixed delay to reward schedule. Under these conditions, administration of SB242084 could have released or enhanced its selection while administration of the agonist WAY161503 reduced its selection as a form of improved response control. Dissociating which of the mechanisms may be controlling behaviour, or whether the resultant effects are a combination of the two ideas, would take further study.

## Supplementary Material

Supporting Methods and Results

## Data accessibility

Primary data will be made available on the Dyrad online platform.
