# Supplementary material for: Feeding behaviour, risk-sensitivity and response control: effects of 5-HT2C receptor manipulations
Source: Philos Trans R Soc Lond B Biol Sci. 2018 Dec 31;374(1766):20180144. doi: 10.1098/rstb.2018.0144 (PMC6335459; doi:10.1098/rstb.2018.0144)
Supplement: Supporting Methods and Results [file rstb20180144supp1.docx]

**Supplementary Information**

**Feeding behaviour, risk-sensitivity and response control; effects of 5-HT_2C_ receptor manipulations**

Trevor Humby^1*^, Yateen Patel^1^, Jenny Carter^1^, Laura-Jean G. Stokes^2^, Robert D. Rogers^2^ and Lawrence S. Wilkinson^1*^

For Philosophical Transactions of the Royal Society B

^1^Behavioral Genetics Group, Schools of Medicine and Psychology, Cardiff University, UK; MRC Centre for Neuropsychiatric Genetics and Genomics and Division of Psychological Medicine and Clinical Neurosciences, School of Medicine, Cardiff University, UK; Neuroscience and Mental Health Research Institute, Cardiff University, UK.

^2^School of Psychology, Bangor University, UK

*to whom correspondence should be addressed:

Dr Trevor Humby, School of Psychology, Cardiff University, Cardiff, CF10 3AT

Tel: +44-(0)29 20870357, Fax: +44-(0)29 20874858, e-mail: [humbyt@cardiff.ac.uk](mailto:humbyt@cardiff.ac.uk)

Professor Lawrence Wilkinson, Cardiff University, Cardiff CF10 3AT

Tel: +44-(0)29 20870357, Fax: +44-(0)29 20874858, e-mail: [wilkinsonL@cardiff.ac.uk](mailto:wilkinsonL@cardiff.ac.uk)

Running title: Pharmacology of feeding behaviour, risk and response control in mouse models

**Supplementary Information**

- Supplementary Methods Figure 1
- Supplementary Methods Table 1
- Supplementary Results Figure 1
- Supplementary Results Figure 2
- Supplementary Results Figure 3
- Supplementary Results Figure 4

| 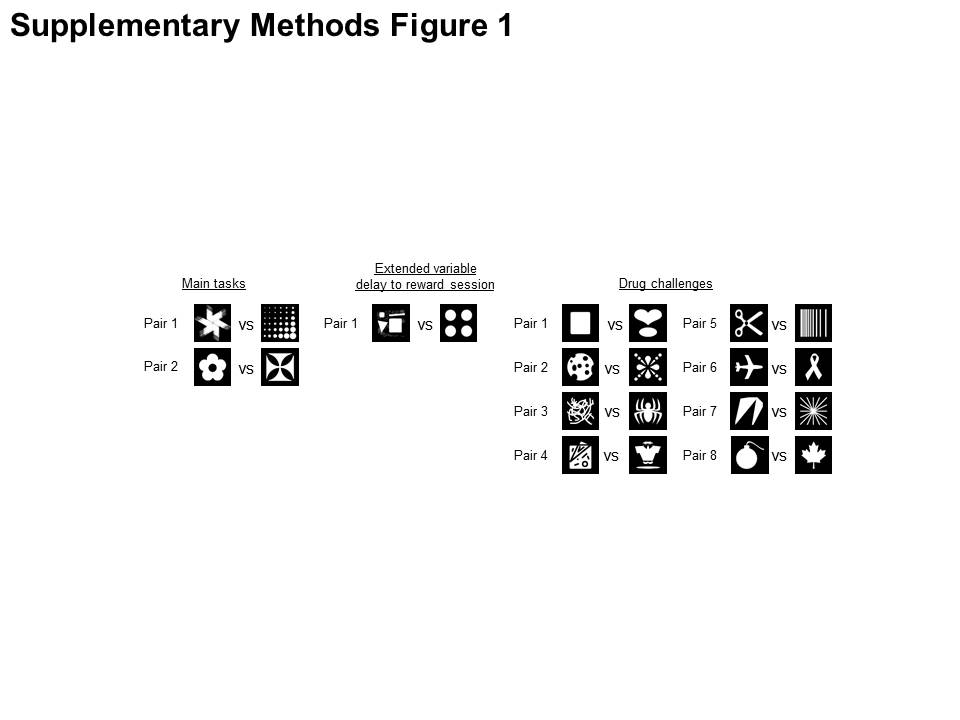 |
| --- |
| **Supplementary Methods Figure 1: Task stimuli.** Different pairs of stimuli were used for each phase of the experiments, with patterns selected for similarity of depth of shading (proportions of black and white) and complexity. For each task, the stimuli associated with each task choice (fixed or variable) were counter-balanced between subjects, and stimuli locations were randomly determined per trial. Patterns were selected from the image database supplied with the touchscreen operant chambers (Campden Cognition, U.K). |

| 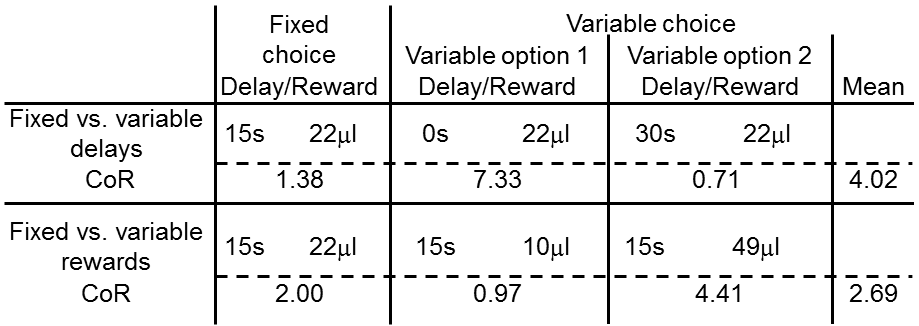 |
| --- |
| **Supplementary Methods Table 1: Task parameters.** The combination of delay durations and reward quantities were determined for each task such that the coefficients of reinforcement were as equivalent as possible [1.2], within the constraints of our previous experience in balancing reward amounts and trial numbers [3,4]. Variable choices were presented with a probability of 0.5 when selected.   1. Kacelnik A, Bateson M. (1997) Risk-sensitivity: crossroads for theories of decision-making. Trends Cogn Sci. 1:304-309. 2. Mazur JE (1997) Choice, delay, probability, and conditioned reinforcement. Anim Learn Behav 25:131-14 3. Humby T, Laird FM, Davies W, Wilkinson LS. (1999) Visuospatial attentional functioning in mice: interactions between cholinergic manipulations and genotype. Eur J Neurosci. 11:2813-2823. 4. Isles AR, Humby T, Walters E, Wilkinson LS. (2004) Common genetic effects on variation in impulsivity and activity in mice. J Neurosci. 24:6733-6740 |

| 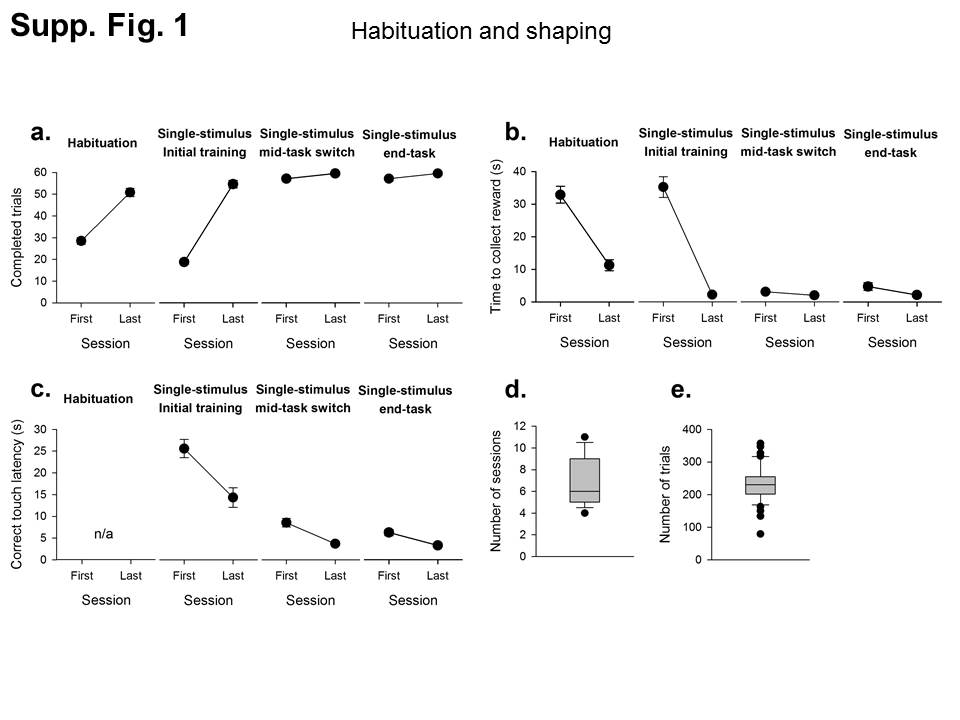 |
| --- |
| **Supplementary Results Figure 1: Habituation and single stimulus shaping.** Mice moved through each of the stages of training with relative alacrity, increasing the number of completed trials by the time each training phase terminated (a). Similarly, response times to collect the food (b) or make contact with a stimulus presented on the touch-screen (c) decreased. Performance for the single-stimulus sessions, used to stabilise responding between the different tasks (mid-task switch, 3 sessions), and at the end of the initial assessment of responding prior to the task manipulation (also 3 sessions) and drug challenges was consistent through each phase of testing (a to c). Subjects took ~7 sessions (d) and ~230 trials (e) to reach criteria when learning to contact the touch-screens to earn reward during the single-stimulus training phase. Data shows mean±SE, N=44. |

| 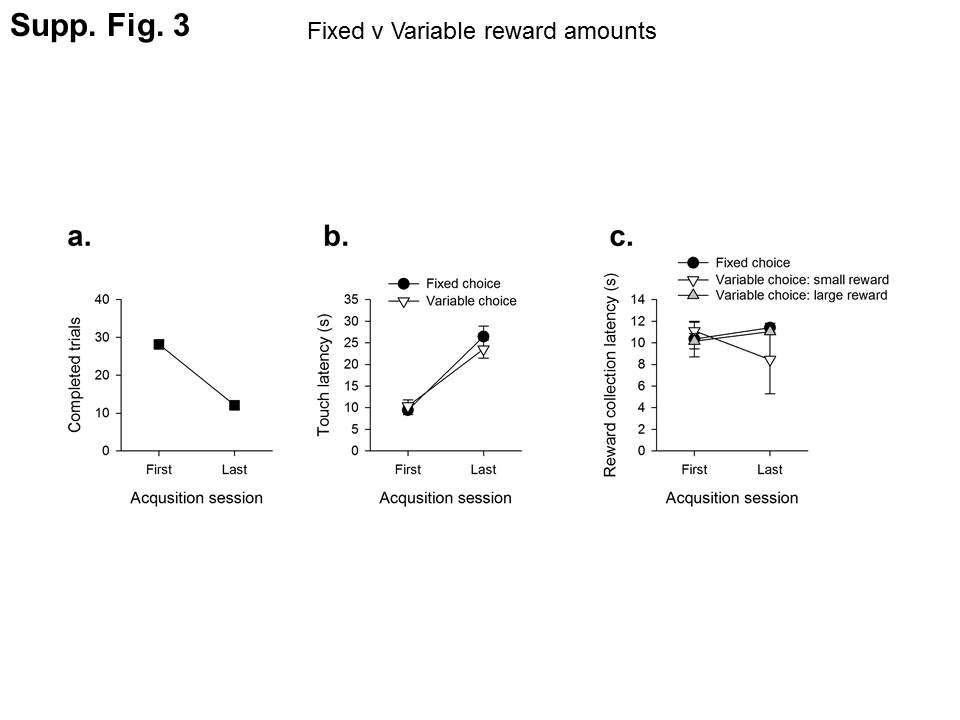 |
| --- |
| **Supplementary Results Figure 2: Additional data for the fixed vs. variable reward amount food schedule task (FST-R).** Data are shown for the initial and last sessions of the initial acquisition of the FST-R task for all mice (N=44). Although two mice did reach the arbitrary 75% performance criterion, their data have been pooled with the main group of mice for clarity. Between the initial session and final session, the number of completed trials decreased (a, t_43_=14.04, p=0.001), and response latencies increased (b, main effect of SESSION, F_1,43_=58.82, p=0.001). There were no differences in the speed of responding between the fixed and variable reward amount choices (main effect of TYPE, F_1,43_=0.71, p=0.41). Reward collection latencies, analysed by ANOVA with within-subject factors of SESSION (First, last)and REWARD AMOUNT (10, 22, 49µl), did not differ during initial acquisition (c, main effect of SESSION, F_1,43_=2.78, p=0.11) or between the different reward amounts presented (main effect of REWARD AMOUNT, F_2,86_=3.01, p=0.07). Data shows mean±SE, N=44. |

| 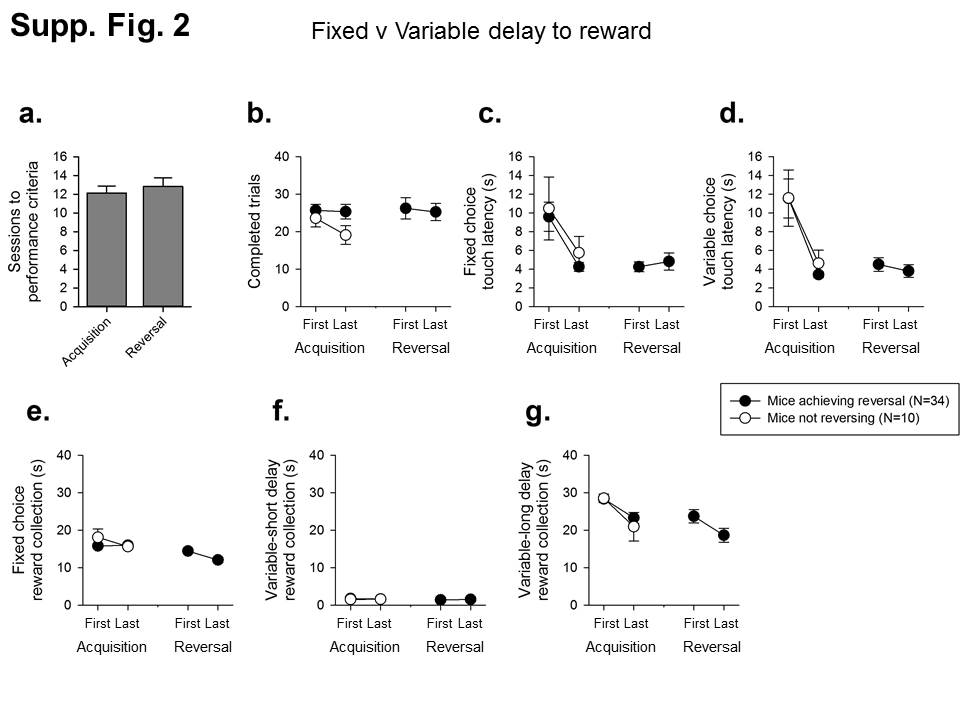 | |
| --- | --- |
| **Supplementary Results Figure 3: Additional data for the fixed vs. variable delay to reward food schedule task (FST-D) task.** Data are shown for the initial and final sessions of the initial acquisition of the FST-D task for mice reaching the arbitrary 75% performance criterion to reverse (34/44) and those which did not meet this criterion (10/44) within 20 sessions. For the mice reaching the performance criterion (34/44), and undergoing reversal, performance in the first and final sessions of the reversal phase is also shown. On average, mice reaching criteria before and after reversal took ~12 sessions to reach performance criteria (a). Data were analysed by ANOVA with between–subject factor of GROUP (reversing, non-reversing mice) and SESSION (first, last). Reaching the performance criteria during the acquisition phase did not affect the trials completed within a session (main effect of GROUP, F_1,42_=1.99, p=0.17), and remained constant throughout this phase (b, main effect of SESSION, F_1,42_=1.54, p=0.22). Post-reversal, there were also no differences in the trials completed from the first to last session (main effect of SESSION, F_1,33_=1.35, p=0.25). There were no significant differences in latency to select either the fixed (c, main effect of GROUP, F_1,42_=0.46, p=0.51) or variable delay to reward choice (d, main effect of GROUP, F_1,42_=0.07, p=0.79) between reversing and non-reversing mice. All mice speeded their responding during initial acquisition (main effect of SESSION, F_1,42_=11.19, p=0.002 and F_1,42_=15.77, p=0.0001, for fixed and variable choice latencies, respectively). Post-reversal, both response latencies remained consistent from the first to last session completed (main effect of SESSION, F_1,33_=0.63, p=0.43, and F_1,33_=1.54, p=0.22, for fixed (c) and variable (d) choice latencies, respectively). As might be expected, the latencies to collect the reward varied dependent on the duration of the delay (e to g), with the smallest latency following reward delivery with the shortest variable delay (0s), and largest collection latency with the long variable delay (30s). However, there were no differences during acquisition between those mice reversing and those that did not for any of the reward collection latencies (main effect of GROUP, F_1,42_=2.96, p=0.11, F_1,42_=0.24, p=0.63 and F_1,42_=0.47, p=0..49, for fixed, short variable and long variable choice delays, respectively). Furthermore, the latencies to collect the reward remained constant from the initial to final sessions before and after reversal for the shorter delays used (main effect of SESSION, F_1,42_=3.54, p=0.07 and F_1,42_=0.33, p=0.86, F_1,33_=3.93, p=0.07 and F_1,33_=0.45, p=0.84, for the fixed and short variable delays, for initial acquisition and reversal respectively). All mice showed a decrease in their latency to collect the reward following the longest delay (g), between the first and last sessions during both initial acquisition (main effect of SESSION, F_1,42_=11.12, p=0.002) and reversal (main effect of SESSION, F_1,33_=4.23, p=0.049). Data shows mean±SE. N=44 for initial acquisition and N=34 for post-reversal. | |
| 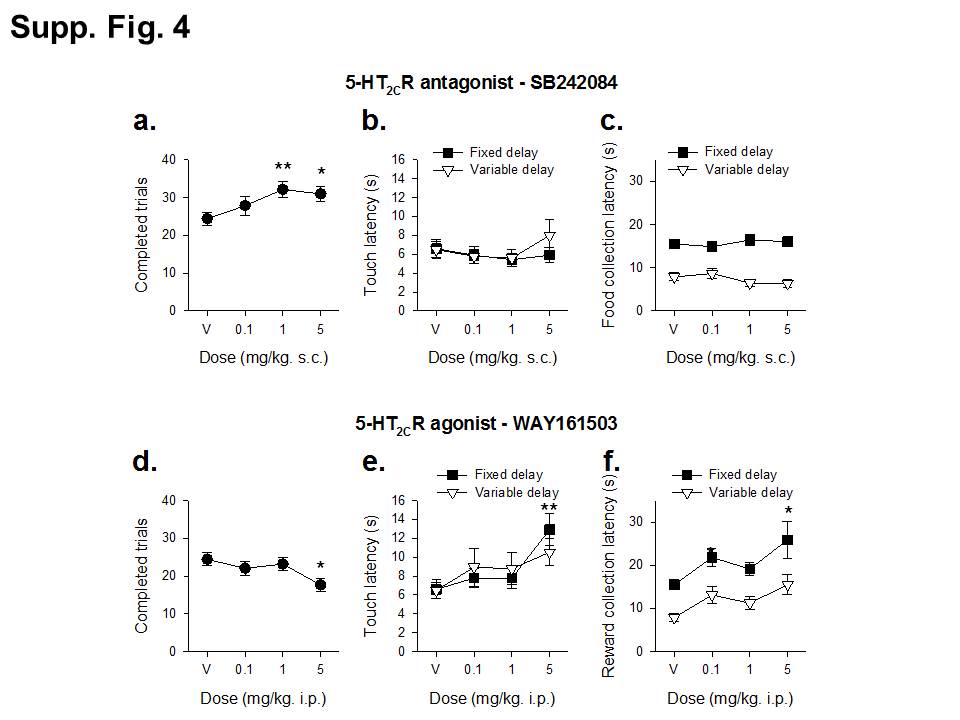 |  |
| **Supplementary Results Figure 4: Additional data for pharmacological manipulations.** Drug challenges were given to mice using the fixed vs. variable delay to reward food schedule task (FST-D) task, with a single session/dose. New stimuli were used in each session. Data were analysed by ANOVA with within–subject factor of DOSE and additional factor of CHOICE (for the comparison between fixed and variable choices). The higher doses of SB242084 significantly increased the number of trials made by the mice (a, main effect of DOSE, F_3,99_=3.17, p=0.03), but did not alter either the latency to choose an option (b, main effect of DOSE, F_3,99_=0.69, p=0.56) or collect the reward (c, main effect of DOSE, F_3,99_=1.05, p=0.37). There were no differences between the latencies to choose the fixed of variable delay to reward options (main effect of CHOICE, F_1,33_=0.80, p=0.39), but mice collected the variable delay to reward option more rapidly than the fixed contingency (main effect of TYPE, F_1,33_=579.93, p=0.0001). Higher doses of WAY161503 decreased the number of trials completed/session (d, main effect of DOSE, F_3,99_=4.08, p=0.008), and increased the latencies to make a choice (e, main effect of DOSE, F_3,99_=4.19, p=0.007) and collect the reward (f, main effect of DOSE, F_3,99_=4.52 p=0.005). There were also no differences between the latencies to choose the fixed of variable delay to reward options (main effect of CHOICE, F_1,33_=0.05, p=0.82), but the latency to collect the reward on fixed delay trials was also greater than for variable delay trials (main effect of CHOICE, F_1,33_=384.78, p=0.0001). Data shows mean±SE, N=34. * and ** denotes p<0.05 and p<0.01, for comparison between a particular drug dose and vehicle, respectively. |  |
